# Model-Based Tool Support for Service Design

**DOI:** 10.1007/978-3-030-45234-6_13

**Published:** 2020-03-13

**Authors:** Francisco J. Pérez-Blanco, Juan M. Vara, Cristian Gómez, Valeria De Castro, Esperanza Marcos

**Affiliations:** 8 University of Paderborn, Paderborn, Germany; 9 ICREA, Open University of Catalonia, Barcelona, Spain; Kybele Research Group, Universidad Rey Juan Carlos, Madrid, Spain

**Keywords:** Service Design, Business Modelling, Model Driven Engineering

## Abstract

This paper introduces a modelling environment for service design that currently supports 5 different notations (Business Model Canvas, e^3^value, Service Blueprint, Process Chain Network and BPMN). Besides, the tool supports the generation of partial views of models based on a particular notation from models made with another one, along with the corresponding relations model.

### 1 Motivation

Born in the context of research on services marketing, service design evolved and gained impact thanks to the promotion of IDEO and it has been eventually established as the entry point to service development for any organization seriously concerned with user experience and digital transformation. For example, take a look at the British government's efforts in this regard, which have been materialized through the Government's Digital Service initiative.

Business modeling is essential in order to achieve a successful service design, since companies need to constantly redesign their business model [3] in their strive towards a successful servitization process. To that end it is key that all the departments of the organization share a clear vision and a common understanding of such models, even when the working languages are different, which, in the case of business models, implies using different notations [4]. Literature reveals indeed that there is a huge number of definitions of what a business model is since the concept has been historically considered from three different perspectives: technology-oriented, strategy-oriented and organization-oriented [2]. Some authors even distinct four categories of business modelling, namely: business process models, business motivation models, business organization models and business rules models [1].

Two of these business modeling disciplines, namely business organization and business process models, are at the core of service design. It implies indeed the use of business models more oriented towards providing a quick and strategic overview of the organization, such as the Business Model Canvas or the e³value model, and business models that are more oriented to show the details of a particular service offering, like Service Blueprints, Process Chain Networks or BPMN models.

Even though tool support is currently available for some of these techniques, there is no comprehensive solution that allows working with all of them. Therefore, different stand-alone tools must be used for each notation, like generic diagramming tools or web-based apps, such as MS-Visio or Lucidchart. Although these can be good options for quick sketching, such tools were not devised to enable later processing of the information gathered in such models [5].

So, provided that the only option to support some notations was to develop a new tool due to the lack of previous tool support (PCN) and the lack of model-based tool support for other notations (Canvas and e³value), a comprehensive toolkit was developed in order to facilitate the building of technological bridges among notations as well as the implementation of post processing tasks, such as validation, autocorrection or model transformations.

To address these problems this report introduces the last version of `INNoVaServ`[1]: a modelling toolkit that comprises a set of visual DSLs implementing different business modeling notations. Regarding previous versions, this one provides tool support for new notations (PCN and BPMN) in the shape of DSLs and it also bundles the tooling needed to register and manage the relationships among business models defined through such DSLs. To that end, the toolkit supports the generation of partial models from models expressed with another notation, along with traces or relations models collecting the relationships between the elements of the models involved in such transformation. In addition to that, `INNoVaServ` supports the formal validation of Service Blueprint and PCN models by means of formal techniques [6, 7] and puts together syntax and semantic checkers for each of the notations supported by the framework.

## 2 Technological Solution

This section first discusses the conceptual architecture of the modelling toolkit introduced in this work to later summarize its development process.

### 2.1 Conceptual Architecture

The conceptual architecture of *INNoVaServ*, which results in a high level of modularization, is illustrated in Figure 1 and can be described according to two orthogonal dimensions.

On the one hand, `INNoVaServ` can be thought of as a set of five integrated DSLs, one for each business modelling notation supported by the tool. This way, in the horizontal dimension of Figure 1 five different modules corresponding to five different DSLs can be distinguished: Business Model Canvas, e³value, Service Blueprint, PCN and BPMN.

On the other hand, the conceptual architecture of `INNoVaServ` leans on the functionality provided by EMF to follow the *separation of concerns* principle [9] by distinguishing the presentation of each model from the model itself. This way, the *presentation* tier includes the components needed to support the edition and representation of models whereas the models are managed by the *logic* tier. As right-hand

[1] http://kybele.es/innovaserv/finalthesis/

side of Figure 1 shows, this distinction corresponds to the usual distinction between the concrete and the abstract syntax of any modelling language.

Besides, a layer connecting the five DSLs is depicted at lower part of Figure 1. Following the idea suggested in [10] to refer to all the tasks related with model management, this layer is called *model processor* and serves as container for different components supporting different model management tasks to be bundled in the tool (validation, weaving, transformation, etc.).

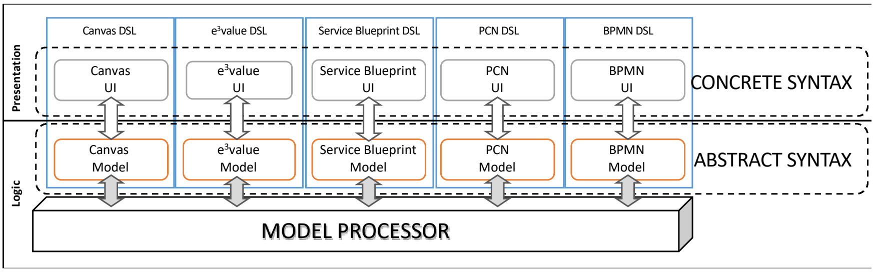

Figure 1. `INNoVaServ` conceptual architecture.

### 2.2 Development Process

Initially, each of the DSLs bundled in `INNoVaServ` were built atop of Eclipse EMF/GMF according to the guidelines sketched in [5] for the development of model-based tools that take the shape of DSL toolkits.

However, due to the recent lack of GMF support, it was necessary the migration of the DSLs from GMF to Sirius. Sirius is also based on Eclipse EMF, and the development process of editors for graphical DSLs with Sirius is still similar to that of GMF: specification of the metamodel; definition of the concrete syntax (Sirius allows to see real time results, besides easily creating different *viewpoints* for the same abstract syntax); identification of the relationships between the models collecting the definition of the abstract and the concrete syntax; creation of the tool palette and finally, manual refinement (if needed) of the generated code.

A series of additional functionalities have been also added to the graphical editors developed, such as the automatic validation and fixing of models using the *Acceleo* language. In addition, to materialize the relationships among the notations supported by the tool, a generic relations (or traces) metamodel (Figure 2) has been defined to support the creation of simplistic relations models and the Epsilon family of languages has been then used to implement a set of model transformations. This way, when any of these transformations runs, a relations model is generated along with the corresponding target model. Specifically, ETL has been used since it supports many-to-many model transformations and it eases the combination of declarative rules with imperative constructions and lazy and greedy rules. This is an essential feature, since many of the model transformations developed are not direct, but require certain level of interaction with the user in order to collect some design decisions that should guide the transformation. In this sense, EOL has been used to improve user interaction by means of dialog boxes and to handle the transformations accordingly.

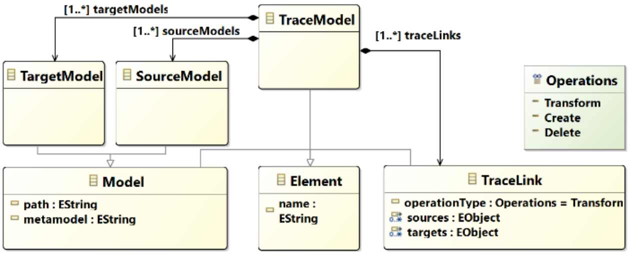

Figure 2. Generic relations metamodel for *INNoVaServ*.

We are conscious of the variety of traces metamodels existing in the literature. Indeed, some of them have been co-authored by us [11]. Nevertheless, a generic simplistic metamodel seemed enough to provide a proof of concept for the proposal. In the meantime, a more complete metamodel, enabling the identification of more sophisticated relationships could be used.

To handle and visualize the information collected in the relationships models, Modelink, a simple yet useful multi-panel editor provided by Epsilon is used. It consists of 2-3 side-by-side EMF tree-based editors, which allows visualizing the source and target models, along with the relations model. Note that relationships collected in the latter can be directly edited in the editor.

Again, it is worth noting that the visualizations provided by Modelink are planned to be improved by developing ad-hoc multi-panel editors like those presented in [11]. For instance, integrated overviews of all the models involved in a given project and their relationships could be supported this way.

Finally, since the toolkit is still basically an EMF/GMF tool, it is consequently interoperable with any other EMF/GMF existing tool. Note that there exists plenty of them since EMF/GMF has turned to be the de-facto standard for the development of model-based tools for the last 10 years. For instance, leaning on Papyrus, UML models could be almost immediately combined with those supported by `INNoVaServ` for Service Design tasks.

### 3 Related Works

This section reviews existing works in the area from both the methodological and technical point of view. However, it is worth noting from the beginning that none of the existing works or tools deal with all the notations supported by `INNoVaServ`, neither provide tool support to enable the processing of the information generated during a service design project.

A quick look at the plenty of systematic literature reviews on business process modelling and the topics covered by them shows that this is somehow the most mature business modelling discipline. Recent reviews are indeed not focused on characterizing existing proposals, since that has been largely done in the past, but on available mechanisms to assess their quality [12] or complexity [13].

However, despite the number of works in the area, still new approaches for business process modelling [15] and BPMN dialects [14] appear every so often. Many of them are focused indeed on shortening the distance between professionals from business areas and business process modelling notations [16].

By contrast, instead of defining yet-another business process modelling language tailored to business professionals, the aim of `INNoVaServ` is at providing them with tool support for the languages they are already using, like the Service Blueprint, BPMN or the PCN. At the same time, providing support to strategy - and organization - oriented business modelling notations like Business Model Canvas or e³value, will help to shorten the historical distance between IT and business practitioners. The models defined and handled by management areas become directly connected (or even mapped) to the models used by IT practitioners, more frequently expressed in terms of BPMN or UML.

On the other hand, despite the recent interest attracted by the field due to the rise of product-service-systems [8], business intelligence modelling [17] and some other disciplines, research on strategic- and organization-oriented business modelling, is still at an early stage, probably because the business process model hype preceded the business model one [4].

Regarding tool-support, provided that no tool has been found supporting the five notations integrated in `INNoVaServ`, some of the existing tools supporting at least two of them are briefly discussed in the following.

*Canvanaizer*[2] and *Real Time Board*[3] are web-based applications that supports collaborative edition of Business Model Canvas and Service Blueprint diagrams. They own a simple and intuitive graphical interface (specially the latter) but they are not based on models, so the represented information is merely graphical. They do not offer export capabilities in a format suitable for post-processing (such as XML), so the output format is reduced to a simple image. Both are commercial solutions, offering free limited editions.

Tool support for e³value was so far limited to the *e³editor*, a desktop application that allows representing graphically and accurately e³value diagrams. Models can be persisted in RDF format, which simplifies export/import tasks.

Regarding PCN, no tool has been found supporting this notation. The only way of defining PCN diagrams to date was using generic diagramming apps or even image editors, like MS Visio or Lucidchart.

Finally, as already mentioned, there are plenty of BPMN tools, such as Bonita Studio, Signavio, BizAgi or IBM WebSphere, each one providing different capabilities.

All this given, to the best of our knowledge this is the first proposal to consider the business modelling notations discussed here and providing tool support to use them in the context of an integrated environment which ease the transition between each of the notations considered.

**Acknowledgements.** This work has been partially funded by the Regional Government of Madrid, through the FORTE-CM project (S2018/TCS-4314) and the Spanish MINECO, through the MADRID project (TIN2017-88557-R).

[2] https://canvanizer.com/
[3] https://realtimeboard.com/
